# Skin cancer and actinic keratosis in people with albinism: a systematic review and meta-analysis^؆^

**DOI:** 10.1016/j.abd.2026.501374

**Published:** 2026-06-27

**Authors:** Fernanda Tranquillini, Vitor Gonçalves Soares, Beatriz Ximenes Mendes, Fnu Krish, Gabrielle Cassulo Franciscatti, Maria Victória Quaresma

**Affiliations:** a Department of Medicine, Universidade de Ribeirão Preto, Guarujá, SP, Brazil; b Department of Medicine, Universidade Federal dos Vales do Jequitinhonha e Mucuri, Diamantina, MG, Brazil; c Department of Medicine, Centro Universitário Christus, Fortaleza, CE, Brazil; d Department of Medicine, Dr. Baba Saheb Ambedkar Medical College and Hospital, Delhi, India; e Department of Dermatology, Faculty of Medicine, Universidade de São Paulo, São Paulo, SP, Brazil; f Department of Dermatology, Faculty of Medicine, Hospital das Clínicas, Universidade de São Paulo, São Paulo, SP, Brazil

**Keywords:** Albinism, Basal cell carcinoma, Carcinoma, squamous cell, Keratosis, actinic, Melanoma, Skin cancer

## Abstract

**Background:**

People with albinism, a genetic condition characterized by reduced melanin production, have an increased risk of developing skin cancer due to their diminished photoprotection. The global prevalence of albinism is estimated at 1 in 17,000 individuals, but it varies significantly by region, being more common in certain parts of Africa. Despite this heightened vulnerability, data on the prevalence of skin cancer and its subtypes in this population remain limited.

**Objective:**

This study aimed to determine the prevalence of Skin Cancer (SC) and Actinic Keratosis (AK) among patients with albinism.

**Methods:**

A systematic search of PubMed, Embase, Web of Science, and Cochrane databases was conducted, only including cross-sectional and cohort studies. The review adhered to Preferred Reporting Items for Systematic Reviews and Meta-Analysis (PRISMA) guidelines. Statistical analyses were performed using R software, and heterogeneity was assessed via the I^2^ statistic.

**Results:**

Among 1,747 individuals with albinism from 12 studies, the pooled prevalence of actinic keratosis (AK) was 38% (95% CI 17.7–60.3; I^2^ = 99%), and that of skin cancer (SC) was 19% (95% CI 13.5–24.4; I^2^ = 89%). Within the SC group, squamous cell carcinoma (SCC) accounted for 55.4% (95% CI 36.2–73.8; I^2^ = 94%), basal cell carcinoma (BCC) for 33.7% (95% CI 19.6–49.2; I^2^ = 91%), and malignant melanoma (MM) for 0% (95% CI 0.00–0.56; I^2^ = 0%).

**Conclusion:**

This study demonstrates a high global prevalence of actinic keratosis (AC) and skin cancer (SC) among individuals with albinism, reinforcing the need for targeted surveillance, preventive strategies, and treatments tailored to this high-risk population. Overall, heterogeneity remained high across outcomes despite the exclusion of individual studies.

**Study limitations:**

The results show persistent heterogeneity driven by differences in study design, diagnostic criteria, and the predominance of cross-sectional data. Future research should aim to address these gaps by employing longitudinal designs, standardizing diagnostic criteria, and including detailed patient-level data.

## Introduction

Albinism is a rare inherited condition characterized by reduced or absent melanin synthesis, leading to hypopigmentation of the skin, hair, and eyes. Its global prevalence is estimated at 1 in 17,000 individuals. The most common form, oculocutaneous albinism (OCA), comprises seven subtypes (OCA1-OCA7), with OCA2 being the most prevalent worldwide, particularly in sub-Saharan Africa.^1^ Less commonly, albinism presents as syndromic forms such as Hermansky-Pudlak and Chediak-Higashi syndromes.^1^

Due to the absence of melanin, individuals with albinism lack natural photoprotection, placing them at significantly increased risk for ultraviolet-induced skin damage and cutaneous malignancies. In regions with limited access to dermatologic care, these cancers often progress undiagnosed and untreated, leading to premature mortality.^2^ Therefore, regular skin monitoring and immediate interventions, along with early diagnostic screening, are crucial for improving the prognosis of skin cancer in patients with albinism.^3^ Additionally, health education for the general population is essential to prevent cancer-promoting factors in individuals with albinism.

Although reliable evidence remains limited, a previous systematic review and meta-analysis assessed the prevalence of squamous and basal cell carcinoma in people with albinism, focusing exclusively on the African population.^4^ Our study expands upon this by incorporating data from a global population and including additional conditions such as malignant melanoma and premalignant lesions, specifically, actinic keratosis. To strengthen methodological rigor, we excluded case reports. These findings aim to support dermatologists in understanding the full spectrum of skin cancers affecting individuals with albinism, while also promoting awareness and developing prevention strategies to reduce the burden of these preventable diseases.

## Material and methods

This systematic review and meta-analysis were reported following the Preferred Reporting Items for Systematic Reviews and Meta-Analysis (PRISMA) guidelines.^5^ The review protocol was registered with the International Prospective Register of Systematic Reviews (PROSPERO),^6^ registration number (CRD42024538147).

### Eligibility criteria

Inclusion criteria:

1) Cross-sectional and cohort studies.
2) Individuals of any age, sex, or ethnicity diagnosed with any type of albinism (including syndromic and non-syndromic forms).
3) Studies reporting quantitative data on at least one of the outcomes of interest (actinic keratosis or skin cancer).
4) Articles published in English, with no restriction on publication date.

Exclusion criteria:

1) Studies including individuals without clinical albinism, including heterozygous carriers.
2) Studies that did not specify the type of skin cancer evaluated.
3) Studies lacking quantitative data on the outcomes of interest.
4) Case reports or case series.
5) Articles not published in English.

### Study strategy and data extraction

We systematically searched PubMed, Cochrane, Embase, and Web of Science databases for studies published up to 2024. The following search strategy was used: (albinism OR albino OR “Hermansky-Pudlak” OR “Chediak-Higashi” OR oculocutaneous) AND (‘skin cancer” OR “skin neoplasm” OR skin neoplasm OR “cutaneous malignancy” OR “skin lesion” OR keratosis OR melanoma). The Medical Subject Headings (MeSH) “skin neoplasm” was used only in the Cochrane database.

Zotero was used for reference management and deduplication of records. Rayyan was used to screen titles and abstracts and assess full-text eligibility. Two authors (B.X.M. and F.T.) independently screened studies based on the predefined inclusion and exclusion criteria. Data were extracted by two authors (B.X.M and F.T.) using a standardized form. Any discrepancies were resolved through discussion and consensus.

### Statistical analysis

For each binary outcome, pooled prevalence estimates with 95% Confidence Intervals (95% CIs) were calculated. Heterogeneity was assessed using the Cochrane *Q*-test and I² statistics. Outcomes were considered to have high heterogeneity when the *Q*-test p-value < 0.10 and I^2^ > 40%. Sensitivity analysis was performed to address high heterogeneity, systematically excluding one study at a time and recalculating the pooled results (leave-one-out analysis). We also performed univariable meta-regression analyses to assess interactions with two covariates (time of follow-up; absolute latitude of the study center) for all outcomes with high heterogeneity. Publication bias was evaluated through funnel plot analysis and Egger’s regression test. All statistical analyses were performed using R software (version 4.3; R Foundation for Statistical Computing, Vienna, Austria).

### Risk of bias assessment

The risk of bias and methodological quality of individual studies were assessed using the Newcastle-Ottawa Scale (NOS) for cohort studies^7^ and a modified version of the NOS for cross-sectional studies, as proposed by Modesti et al.^8^ For cohort studies, the NOS evaluates three domains: selection of study groups, comparability of groups, and ascertainment of outcomes (Supplementary Table 1). For cross-sectional studies, the modified NOS includes analogous domains adapted to observational design (Supplementary Table 2).^9^, ^10^, ^11^, ^12^, ^13^, ^14^, ^15^, ^16^, ^17^, ^18^, ^19^, ^20^

Each item was rated with a star if the study met predefined quality criteria, with a maximum score of 9 stars for cohort studies and 10 for cross-sectional studies (Supplementary Tables 1 and 2). Publication bias was assessed through visual inspection of funnel plots for outcomes with at least ten included studies, by plotting individual study weights against their corresponding effect estimates.

## Results

### Study selection and characteristics

**Figure 1:**
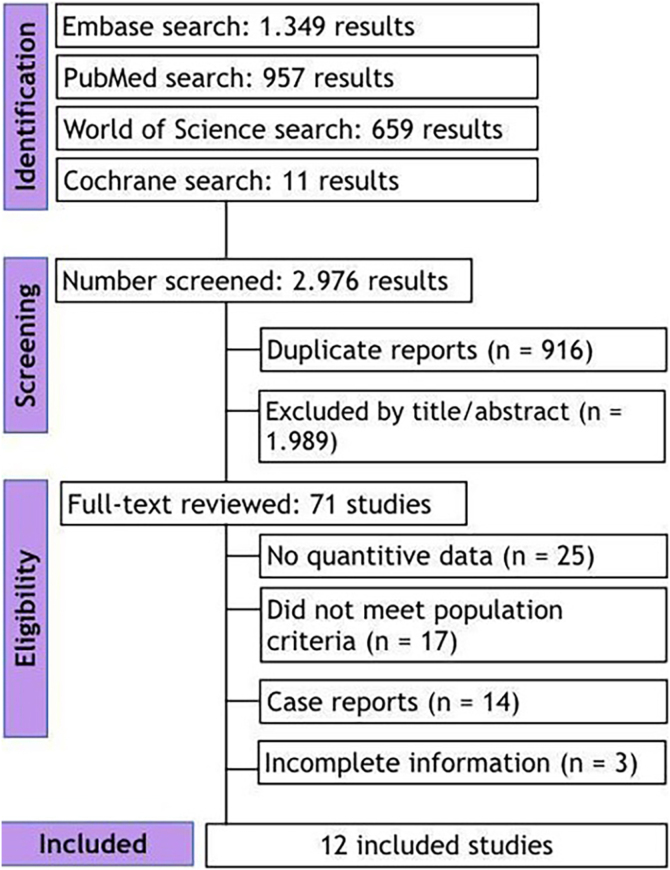
PRISMA flow diagram of study screening and selection.

The search strategy identified 2,976 results. After removing duplicates and studies that were not related to the topic of interest based on title and abstract, 71 studies were selected for full-text review. Of these, 59 did not meet the study’s criteria (Fig. 1). A total of 12 studies were included, comprising 1,747 individuals with albinism.^9^, ^10^, ^11^, ^12^, ^13^, ^14^, ^15^, ^16^, ^17^, ^18^, ^19^, ^20^ The mean average age across studies was 37.43-years, and the overall male-to-female ratio was 0.8:1. The remaining baseline characteristics of the included studies are in Table 1.^9^, ^10^, ^11^, ^12^, ^13^, ^14^, ^15^, ^16^, ^17^, ^18^, ^19^, ^20^

**Table 1:** Baseline characteristics of included studies.

Study	Study design	Follow up^b^	Country	N of albinos		N of albinos with skin cancer			N of skin cancers (in situ included)				
				Total	With AK (%)	Total (%)	Male (%)	Age^a^	Total	BCC (%)	SCC (%)	MM (%)	Others (%)
Zongo, 2023^9^	CS	9	Burkina Faso	139	11 (8)	46 (33)	16 (35)	36/9	71	0 (0)	71 (100)	0 (0)	0 (0)
Malave, 2022^10^	CS	‒	Puerto Rico	29	13 (45)	7 (24)	‒	‒	7	4 (57)	2 (29)	0 (0)	1 (14)
Hassan, 2022^11^	CS	1	Haiti	106	60 (57)	31 (29)	11 (35)	‒	31	10 (32)	12 (39)	0 (0)	9 (29)
Mouhari-Toure, 2021^12^	CS	2	Togo	517	298 (58)	64 (12)	31 (48)	40/16	137	63 (46)	67 (49)	2 (2)	0 (0)
Ramos, 2021^13^	CS	1	Brazil	74	32 (43)	16 (22)	‒	38/15	16	8 (50)	7 (44)	1 (6)	0 (0)
Inena, 2020^14^	CS	3	D.R.C.	205	205 (100)	61 (30)	32 (53)	27/12	28	10 (36)	18 (64)	0 (0)	0 (0)
Enechukwu, 2020^15^	CS	‒	Nigeria	90	34 (38)	18 (20)	9 (50)	39/4	58	22 (38)	9 (16)	0 (0)	9 (16)
Marçon, 2019^16^	CS	7	Brazil	146	75 (51)	38 (26)	15 (40)	47/18	35	18 (51)	15 (43)	2 (6)	0 (0)
Emadi, 2017^17^	PC	2	Kenya	151	56 (37)	20 (13)	13 (65)	‒	20	12 (60)	8 (40)	0 (0)	0 (0)
Toro, 1999^18^	CS	3	USA	65	‒	6 (9)	‒	‒	6	3 (50)	3 (50)	0 (0)	0 (0)
Bothwell, 1997^19^	CS	‒	S.A.	61	‒	5 (8)	‒	‒	5	0 (0)	5 (100)	0 (0)	0 (0)
Lookingbill, 1995^20^	CS	‒	Tanzania	164	69 (42)	10 (6)	5 (50)	35/13	9	2 (22)	6 (66)	0 (0)	1 (11)

a Mean in years; ‒ Not informed; SD, Standard Deviation; AK, Actinic Keratosis; BCC, Basal Cell Carcinoma; SCC, Squamous Cell Carcinoma; MM, Malignant Melanoma; CS, Cross-Sectional; PC, Prospective Cohort.

b Years in which the study was conducted; N, Number.

### Pooled analysis of all studies

A. Actinic keratosis

**Figure 2:**
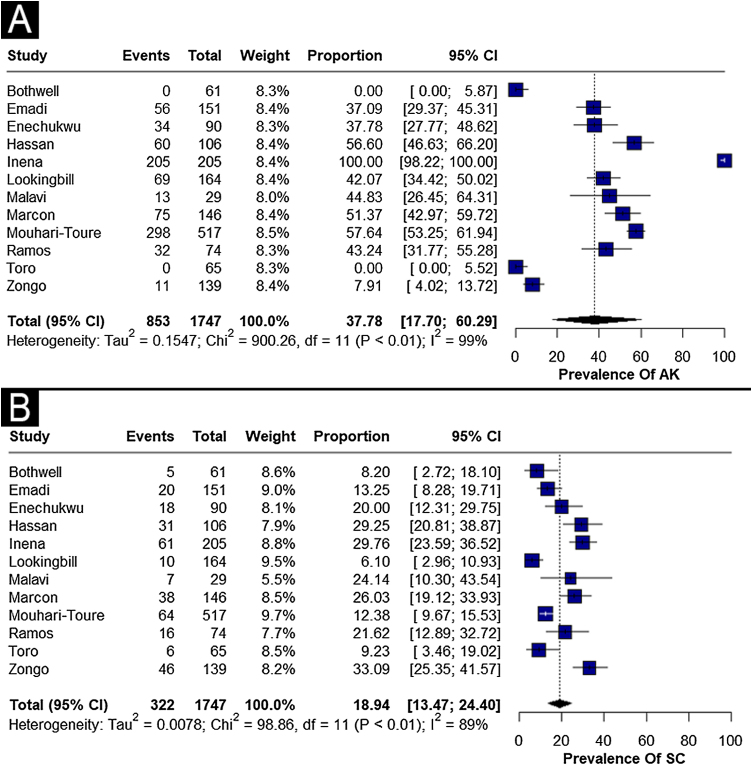
(A) Prevalence of Actinic Keratosis (AK) in people with albinism. (B) Prevalence of Skin Cancer (SC) in people with albinism.

Twelve studies with a total of 1,747 patients were included for the AK prevalence analysis. The pooled prevalence of AK among individuals with albinism was 37.78% (95% CI 17.70‒60.29%; I^2^ = 99%; Fig. 2A), using a random-effects model.

B. Skin cancer

Twelve studies with a total of 1,747 patients were included for the analysis of the prevalence of SC. The pooled prevalence of SC was 18.94% (95% CI 13.47–24.4%; I^2^ = 89%; Fig. 2B).

B.1. Basal cell carcinoma

**Figure 3:**
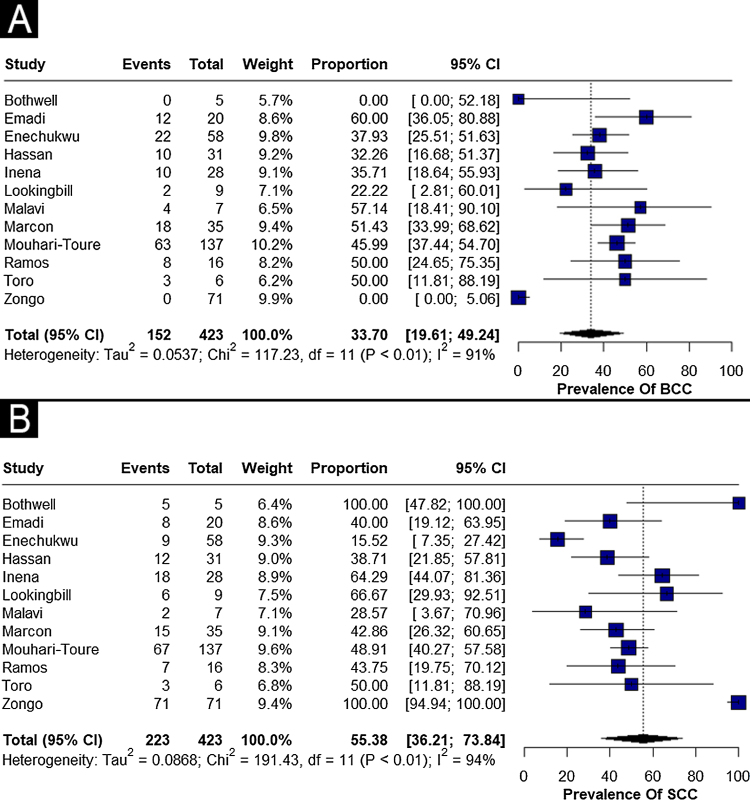
(A) Prevalence of Basal Cell Carcinoma (BCC) among individuals with albinism with SC. (B) Prevalence of Squamous Cell Carcinoma (SCC) among individuals with albinism diagnosed with SC.

Twelve studies with a total of 423 biopsies were included for the analysis of the prevalence of BCC. The prevalence of BCC was 33.7% (95% CI 19.61%–49.24%; I^2^ = 91%; Fig. 3A).

B.2. Squamous cell carcinoma

Twelve studies with a total of 423 biopsies were included for the analysis of the prevalence of SCC. The prevalence of SCC was 55.38% (95% CI 36.21%–73.84%; I^2^ = 94%; Fig. 3B).

### Malignant melanoma

**Figure 4:**
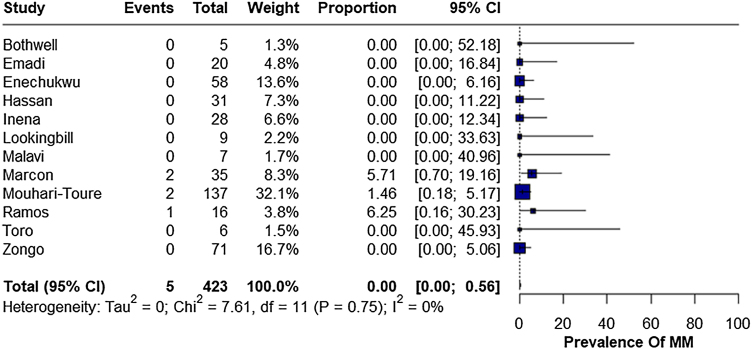
Prevalence of Malignant Melanoma (MM) among individuals with albinism diagnosed with SC.

Twelve studies with a total of 423 biopsies were included for the analysis of the prevalence of MM. The pooled prevalence of MM was 0.0% (95% CI 0.00%–0.56%; I^2^ = 0%; Fig. 4).

### Leave-one-out analysis

Leave-one-out analyses were conducted for all outcomes, except for MM, as its heterogeneity was below 40%. For the outcomes of BCC, SC, and SCC, the omission of the Zongo et al. study resulted in the lowest observed heterogeneity (I^2^ = 30%, 87%, and 75%, respectively; Supplementary Figs. 1‒3). For AK, exclusion of the Inena et al. study reduced heterogeneity to its lowest value (I^2^ = 97%; Supplementary Fig. 4). Overall, heterogeneity remained high across outcomes despite the exclusion of individual studies.

### Meta-regression analysis

Meta-regression was conducted for the outcomes of AK, BCC, SC, and SCC. The more distant a study center was from the equator (the greater its absolute latitude), the lower its incidence of AK (b: -0.02; 95% CI -0.05 to 0.00; p-value = 0.047; R^2^ = 21.46%) (Table 2). However, higher follow-ups were paradoxically associated with less BCC (b: -0.06; 95% CI -0.12 to -0.01; p-value = 0.031; R^2^ = 38.50%), but more SCC (b: -0.07; 95% CI 0.02 to 0.13; p-value = 0.007; R^2^ = 51.30%) (Table 2). Overall, follow-up and latitude had no significant impact on most of the outcomes, but for those outcomes in which they had a significant impact, they explained the study's variation from 21.46% to 51.30% (Table 2).

**Table 2:** Meta-regression.

Outcome	Predictor	b (95% CI)	p-value	R^2^
AK	Follow-up	-0.04 (-0.16 to 0.07)	0.454	0
	Latitude	-0.02 (-0.05 to 0)	0.047^a^	21.46
SC	Follow-up	0.02 (-0.01 to 0.04)	0.175	14.40
	Latitude	0 (-0.01 to 0.01)	0.887	0
BCC	Follow-up	-0.06 (-0.12 to -0.01)	0.031^a^	38.50
	Latitude	0 (-0.02 to 0.01)	0.67	0
SCC	Follow-up	0.07 (0.02 to 0.13)	0.007^a^	51.30
	Latitude	0.01 (-0.02 to 0.03)	0.638	0

Meta Regression for the outcomes of AK, BCC, SC, and SCC using time of Follow-up and approximate absolute Latitude of the study center(s). b, Partial unstandardized regression coefficient for each predictor; CI, Confidence Interval; R^2^, Proportion of variance accounted by the predictor. ^a^ p-value < 0.05.

### Quality assessment

The cohort study by Emandi et al. had a score of 4/8. The cross-sectional study by Malave et al. had a score of 4/10, whereas the other 10 cross-sectional studies had a score of over 4/10 and under 7/10. All studies lost points at the Selection criteria. For cross-sectional studies, this was due to no description of the measurement tool.

Publication bias was investigated for all outcomes, as at least 10 studies were available for each. The visual inspection of the funnel plots shows some asymmetry for the outcomes of MM and SCC (Supplementary Figs. 5‒6). No asymmetry is visually detected for the outcomes of AK, BCC, and SC (Supplementary Figs. 7‒9). However, this result is discouraged by the Egger’s Test, as a p-value < 0.05 was shown only for the outcome of SC (Table 3).

**Table 3:** Egger’s test.

Outcome	Intercept	LB	UB	*t*	p
BCC	0.589	-3.693	4.87	0.269	0.793
AK	-11.011	-24.838	2.815	-1.561	0.15
SC	4.104	0.754	7.454	2.401	0.037
MM	0.653	-0.364	1.671	1.258	0.237
SCC	-0.43	-5.914	5.055	-0.154	0.881

LB, Lower Bound of the intercept; UB, Upper Bound of the intercept; *t*, *t*-value of the Egger's test; p, p-value of the Egger's test.

## Discussion

In this meta-analysis, a total of twelve studies comprising 1747 patients were included. The primary objective of this study was to estimate the prevalence of skin cancer among individuals with albinism. The second objective of our study was to identify the most prevalent type of skin cancer in this population. The main findings of this systematic review and meta-analysis include: 1) The prevalence of skin cancer is high in individuals with albinism and more pronounced than in other populations; 2) Skin cancer occurs at an earlier age in people with albinism compared with the general population; 3) SCC is the most prevalent type of SC in this group; 4) There is a high prevalence of AK in people with albinism; and 5) Individuals in developing countries have higher rates of SC than those in developed countries. While skin cancer is a significant public health concern globally for many populations, its impact on individuals with albinism is particularly alarming.

These findings are underscored by broader epidemiological data on SC prevalence in different populations. Approximately 20%–30% of all malignancies in the Caucasian population are SC, making it the most prevalent cancer in this community. In contrast, SC accounts for just 1%‒2% of cancer cases in individuals with more pigmented skin.^3^, ^21^ This disparity highlights the heightened vulnerability of individuals with lighter skin tones to skin cancer, especially those with albinism. Our meta-analysis aligns with these statistics, revealing a pooled skin cancer prevalence of 18.9% among individuals with albinism. According to the United Nations, a significant proportion of individuals with albinism in sub-Saharan Africa die prematurely from preventable causes such as skin cancer, with many not reaching the age of 40.^3^, ^21^ Therefore, it is crucial to understand the prevalence of each type of SC in this population.

In addition to the high prevalence, another critical aspect is the early onset of skin cancer in individuals with albinism. Our findings confirm that skin cancer tends to occur at significantly younger ages in this population, with mean ages ranging from 27- to 47-years old. This trend is consistent with studies conducted in Nigeria, where most individuals with albinism were found to be under 30-years of age.^22^, ^23^ In contrast, the average age at diagnosis in the general population typically ranges from 60- to 74-years old.^22^, ^24^ However, this finding may reflect the reduced life expectancy observed in individuals with albinism, particularly in underserved regions.

Beyond age, identifying the most common types of skin cancer in individuals with albinism provides further insight into their specific health risks. A previous meta-analysis encompassing African countries found that SCC was the most common type of skin cancer among individuals with albinism, with a prevalence of 64%, followed by BCC at 31%.^25^ Although our meta-analysis includes global data, our results agree with this finding, showing a SCC prevalence of 55%, followed by a BCC prevalence of 33%.

Isolated cases of malignant melanoma were identified in three studies; however, the pooled prevalence was 0.0%, with a 95% Confidence Interval ranging from 0.00% to 0.56%. This result is not statistically significant, and the presence of melanoma cannot be confirmed based on the pooled estimate. Nevertheless, the possibility of rare occurrences cannot be entirely excluded, supporting the inclusion of melanoma in dermatologic surveillance of individuals with albinism.

Given the high prevalence of SCC and BCC, it is important to understand the conditions that contribute to these cancers. Actinic keratosis (AK) is a key consideration in albinism due to its potential for malignant transformation.^25^ Our meta-analysis revealed a substantial prevalence of AK (37%) within this population, aligning with prior studies. For instance, one Australian study reported that 59% of SCC cases developed from a pre-existing AK lesion within one year.^26^

This underscores the importance of early detection and treatment of AK to prevent progression to invasive skin cancers.

While albinism is a global condition, its incidence varies significantly, with higher rates observed in African countries.^27^, ^28^ Factors such as limited geographic mobility and consanguinity, often associated with traditional marriage practices in under-resourced regions, contribute to this increased prevalence.^24^ Concerning Skin Cancer (SC), although developed countries report higher incidence rates, the majority of skin cancer-related deaths occur in developing nations.^29^ Sub-Saharan African countries, in particular, face disproportionately high rates of premature cancer mortality and limited healthcare infrastructure.^30^ These disparities raise critical concerns regarding the prevention and treatment of skin cancer in individuals with albinism.

Most included studies lacked information on individual-level factors that influence skin cancer risk, such as occupational sun exposure, sunscreen use, and other protective behaviors. The absence of these data limits our ability to evaluate behavioral contributors to cancer development and hinders tailored prevention strategies.

Individuals with albinism, particularly in sub-Saharan Africa, often face profound stigma and social marginalization. In some communities, they are perceived as cursed or spiritually different, leading to discriminatory practices that can include ostracism, violence, and denial of care. These sociocultural barriers further restrict access to already scarce dermatologic services and preventive interventions.

Despite rigorous methodology, this meta-analysis exhibited substantial heterogeneity, which persisted even after applying funnel plot inspection, Egger’s test, leave-one-out sensitivity analysis, and meta-regression. This variability likely reflects differences in study design, sample size, and diagnostic criteria – particularly for actinic keratosis and non-melanoma cancers, which were not always confirmed histopathologically. Additionally, the predominance of cross-sectional studies limited the ability to assess lesion progression over time. While these factors warrant cautious interpretation, the consistency of findings across multiple studies and the use of robust statistical approaches support the overall validity and relevance of results.

Nonetheless, this study has notable strengths. The inclusion of a substantial number of studies and patients enhanced the statistical power of the analysis. The incorporation of data from various regions and multiple skin cancer types contributes to the external validity of the findings.

Future research should aim to address these gaps by employing longitudinal designs, standardizing diagnostic criteria, and including detailed patient-level data such as sun exposure history, sunscreen use, and socioeconomic indicators. Such efforts will be essential to refine risk stratification and develop more effective screening and prevention strategies tailored to individuals with albinism.

## Conclusion

This meta-analysis demonstrates a high prevalence of skin cancer and actinic keratosis among individuals with albinism, with squamous cell carcinoma being the most frequent subtype. Although malignant melanoma was rarely reported, its inclusion in dermatologic surveillance remains warranted. Skin cancer in this population tends to occur earlier than in the general population, reflecting both biological vulnerability and limited access to preventive care.

Despite substantial heterogeneity and methodological variability across studies, the findings offer valuable insight into a globally neglected public health issue. The lack of individual-level data and the underrepresentation of non-African regions limit the generalizability of results, but highlight critical gaps that should guide future research.

These results underscore the urgent need for targeted prevention strategies, public health interventions, and equitable access to dermatologic services for individuals with albinism, particularly in resource-limited settings.

## ORCID ID

Vitor Gonçalves Soares: 0009-0007-2868-5389

Beatriz Ximenes Mendes: 0009-0009-0788-130X

Fnu Krish: 0009-0006-7606-0511

Gabrielle Cassulo Franciscatti: 0009-0007-7216-0045

Maria Victória Quaresma: 0000-0003-2891-1650

## Research data availability

The entire dataset supporting the results of this study was published in this article.

## Financial support

The authors did not receive support from any organization for the submitted work.

## Authors’ contributions

Fernanda Tranquillini: Conceptualization; study design; data collection; writing (original draft); writing (review and editing).

Vitor Gonçalves Soares: Data analysis.

Beatriz Ximenes Mendes: Data collection.

Fnu Krish: Risk of bias and writing (original draft).

Gabrielle Cassulo Franciscatti: Writing (review and editing).

Maria Victória Quaresma: Writing (review and editing).

## Conflicts of interest

None declared.
