# Supplementary material for: Skin cancer and actinic keratosis in people with albinism: a systematic review and meta-analysis
Source: An Bras Dermatol. 2026 Jun 27;101(4):501374. doi: 10.1016/j.abd.2026.501374 (PMC13321011; doi:10.1016/j.abd.2026.501374)
Supplement: Supplementary file 1 [file mmc1.docx]

ABD-D-25-00695_Supplementary Material

**Supplementary Table 1** Critical appraisal of individual studies according to the Newcastle-Ottawa Scale (NOS) for assessing risk of bias in cohort and crossover studies.

|  | **NOS scale for cohort studies** | | | | | | | | |
| --- | --- | --- | --- | --- | --- | --- | --- | --- | --- |
| **Cohort** | **Selection** | | | | **Comparability** | **Outcome** | | |  |
| **Article** | **1** | **2** | **3** | **4** | **1** | **1** | **2** | **3** | **Score** |
| Emandi et al. | b | c | d | b | c | a | a | a | 4/8 |

Selection 1, Representativeness of the exposed cohort; Selection 2, Selection of the non-exposed cohort; Selection 3, Ascertainment of exposure; Selection 4, Demonstration that the outcome of interest was not present at start of study. Comparability 1, Comparability of cohorts on the basis of the design or analysis. Outcome 1, Assessment of outcome; Outcome 2, Was follow-up long enough for outcomes to occur; Outcome 3, Adequacy of follow up of cohorts.

**Supplementary Table 2** Modified NOS scale for cross-sectional studies by Modesti et al.[9-20]

| **Cross-sectional** | **Selection** | | | | **Comparability** | **Outcome** | |  |
| --- | --- | --- | --- | --- | --- | --- | --- | --- |
| **Article** | **1** | **2** | **3** | **4** | **1** | **1** | **2** | **Score** |
| Zongo et al.[9] | b | b | a | c | a | a | a | 6/10 |
| Ramos et al.[13] | b | a | b | c | a | a | a | 6/10 |
| Toro et al.[18] | a | a | c | c | a | a | a | 6/10 |
| Enechukwu et al.[15] | b | b | c | c | a | a | a | 5/10 |
| Inena et al.[14] | b | a | c | c | a | a | b | 5/10 |
| Malave et al.[10] | b | a | c | c | a | c | a | 4/10 |
| Hassan et al.[11] | b | a | c | c | a | a | a | 6/10 |
| Lookingbill et al.[20] | a | b | c | c | a | a | b | 5/10 |
| Marcon et al.[16] | a | a | c | c | a | b | a | 6/10 |
| Toure et al.[12] | b | b | c | c | a | a | a | 5/10 |
| Bothwell et al.[19] | b | b | c | c | a | a | a | 5/10 |

Selection 1, Representativeness of the sample; Selection 2, Sample size; Selection 3, Non-respondents; Selection 4: Ascertainment of the exposure. Comparability 1, The subjects in different outcome groups are comparable, based on the study design or analysis. Confounding factors are controlled. Outcome 1, Assessment of outcome; Outcome 2, Statistical test.

**Supplementary Figure 1** Leave-one-out analysis for the outcome of BCC. CI, Confidence Interval.


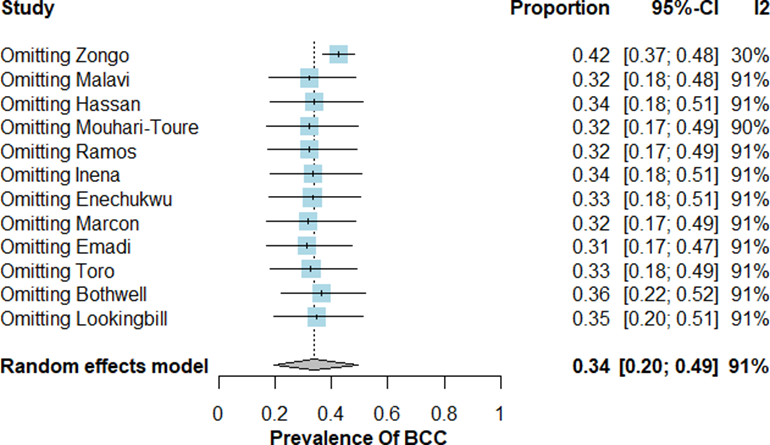


**Supplementary Figure 2** Leave-one-out analysis for the outcome of SC. CI, Confidence Interval.


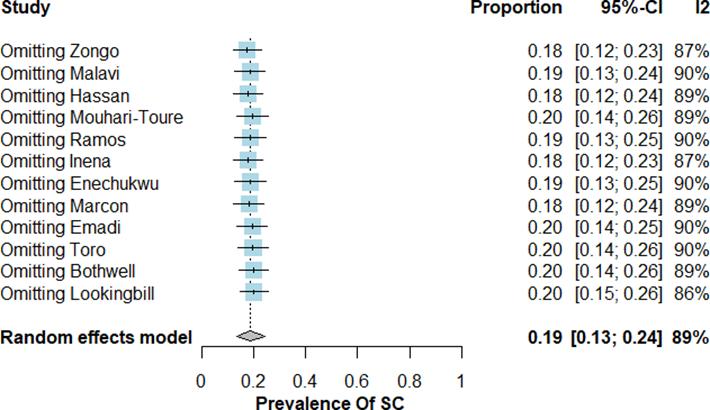


**Supplementary Figure 3** Leave-one-out analysis for the outcome of SCC. CI, Confidence Interval.


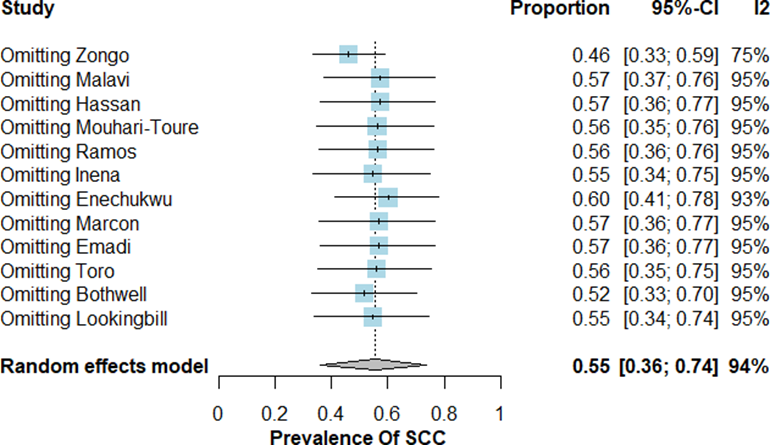


**Supplementary Figure 4** Leave-one-out analysis for the outcome of AK. CI, Confidence Interval.


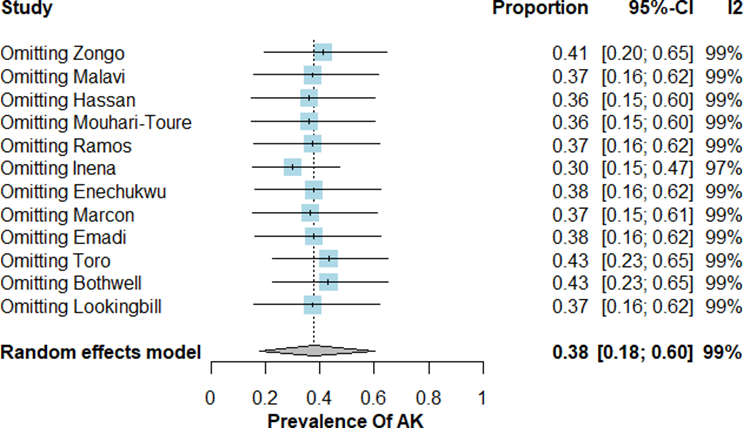


**Supplementary Figure 5** Funnel plot for the outcome of MM.


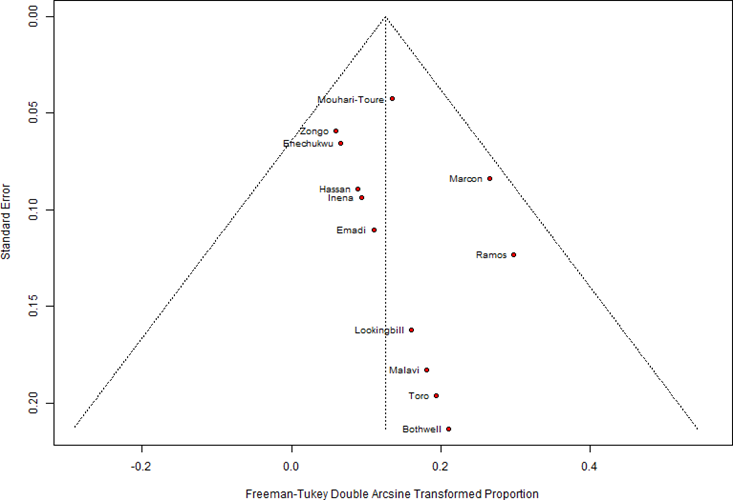


**Supplementary Figure 6** Funnel plot for the outcome of SCC.


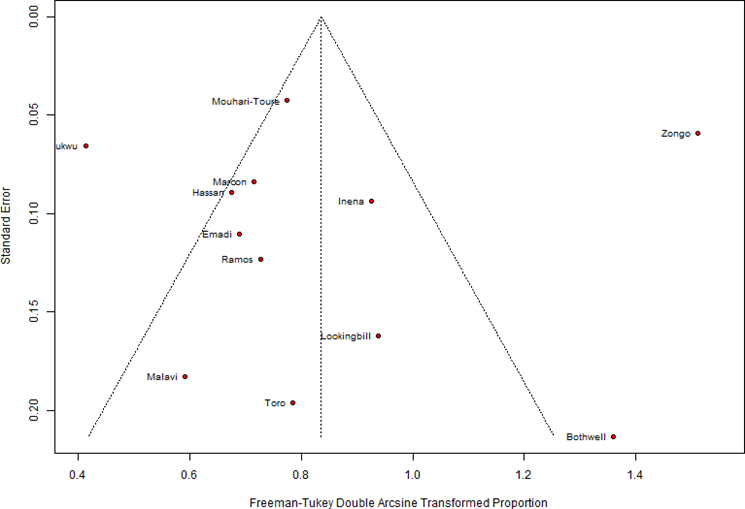


**Supplementary Figure 7** Funnel plot for the outcome of AK.


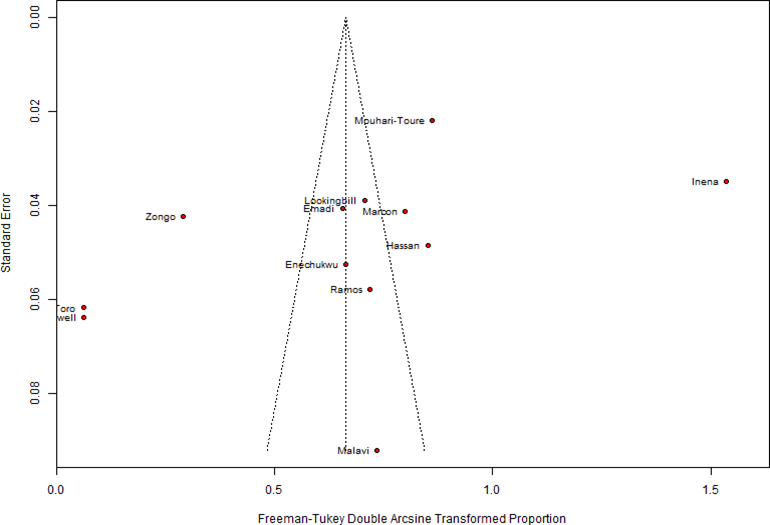


**Supplementary Figure 8** Funnel plot for the outcome of BCC.


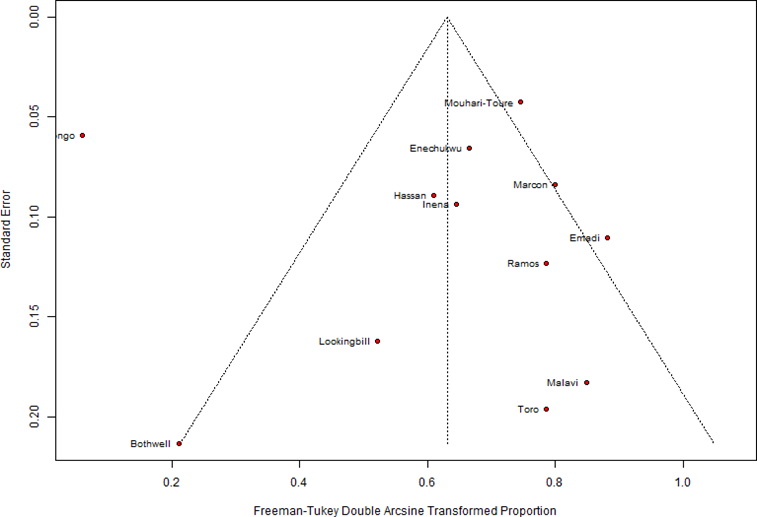


**Supplementary Figure 9** Funnel plot for the outcome of SC.


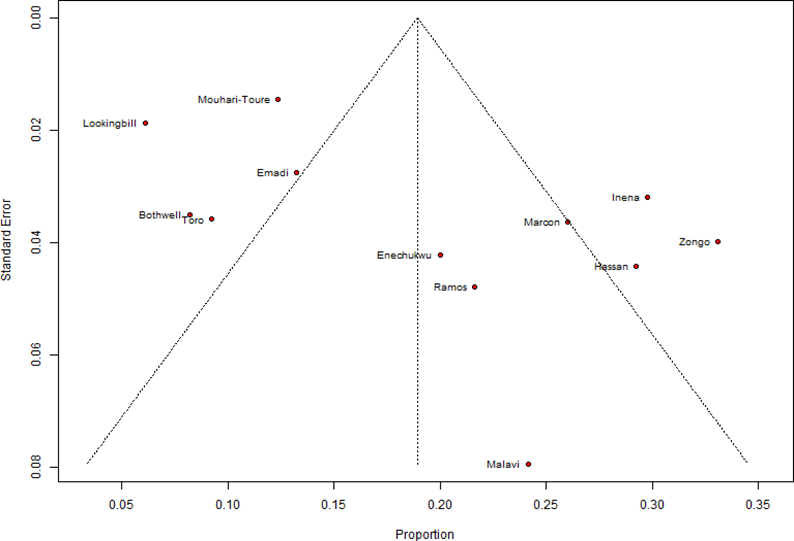


| **Modified NOS scale for cross-sectional studies by Modesti et al.** |
| --- |
|  |
| **Selection:** |
| 1) Representativeness of the sample: |
| a) Truly representative of the average in the target population. * (all subjects or random sampling) |
| b) Somewhat representative of the average in the target population. * (nonrandom sampling) |
| c) Selected group of users. |
| d) No description of the sampling strategy. |
|  |
| 2) Sample size: |
| a) Justified and satisfactory. * |
| b) Not justified. |
|  |
| 3) Non-respondents: |
| a) Comparability between respondents and non-respondents’ characteristics is established, and the response rate is satisfactory. * |
| b) The response rate is unsatisfactory, or the comparability between respondents and non-respondents is unsatisfactory. |
| c) No description of the response rate or the characteristics of the responders and the non-responders. |
|  |
| 4) Ascertainment of the exposure (risk factor): |
| a) Validated measurement tool. ** |
| b) Non-validated measurement tool, but the tool is available or described. * |
| c) No description of the measurement tool. |
|  |
| **Comparability:** (Maximum 2 stars) |
| 1) The subjects in different outcome groups are comparable, based on the study design or analysis. |
| Confounding factors are controlled. |
| a) The study controls for the most important factor (select one). * |
| b) The study control for any additional factor. * |
|  |
| **Outcome:** (Maximum 3 stars) |
| 1) Assessment of the outcome: |
| a) Independent blind assessment. |
| ** b) Record linkage. |
| ** c) Self report. |
| * d) No description. |
|  |
| 2) Statistical test: |
| a) The statistical test used to analyze the data is clearly described and appropriate, and the measurement of the association is presented, including confidence intervals and the probability level (p-value). * |
| b) The statistical test is not appropriate, not described or incomplete. |
|  |
| **NOS for cohort studies** |
|  |
| **Selection:** |
| 1) Representativeness of the exposed cohort |
| a) Truly representative (one star) |
| b) Somewhat representative (one star) |
| c) Selected group |
| d) No description of the derivation of the cohort |
|  |
| 2) Selection of the non-exposed cohort |
| a) Drawn from the same community as the exposed cohort (one star) |
| b) Drawn from a different source |
| c) No description of the derivation of the non-exposed cohort |
|  |
| 3) Ascertainment of exposure: |
| a) Secure record (e.g., surgical record) (one star) |
| b) Structured interview (one star) |
| c) Written self-report; d) No description; e) Other |
|  |
| 4) Demonstration that outcome of interest was not present at start of study: |
| a) Yes (one star) |
| b) No |
|  |
| **Comparability:** |
| 1) Comparability of cohorts on the basis of the design or analysis controlled for confounders: |
| a) The study controls for age, sex and marital status (one star) |
| b) Study controls for other factors (list) (one star) |
| c) Cohorts are not comparable on the basis of the design or analysis controlled for confounders |
|  |
| **Outcome:** |
| 1) Assessment of outcome: |
| a) Independent blind assessment (one star) |
| b) Record linkage (one star) |
| c) Self report |
| d) No description |
| e) Other |
|  |
| 2) Was follow-up long enough for outcomes to occur: |
| a) Yes (one star) |
| b) No Indicate the median duration of follow-up and a brief rationale for the assessment above: |
|  |
| 3) Adequacy of follow-up of cohorts: |
| a) Complete follow up- all subject accounted for (one star) |
| b) Subjects lost to follow up unlikely to introduce bias- number lost less than or equal to 20% or description of those lost suggested no different from those followed. (one star) |
| c) Follow up rate less than 80% and no description of those lost |
| d) No statement |
